# DNA Polymerase δ Is Required for Early Mammalian Embryogenesis

**DOI:** 10.1371/journal.pone.0004184

**Published:** 2009-01-15

**Authors:** Arikuni Uchimura, Yuko Hidaka, Takahiro Hirabayashi, Masumi Hirabayashi, Takeshi Yagi

**Affiliations:** 1 Graduate School of Frontier Biosciences, Osaka University, Suita, Osaka, Japan; 2 Neo-Morgan Laboratory Inc., Tokyo, Japan; 3 National Institute for Physiological Sciences, Okazaki, Aichi, Japan; University of Minnesota, United States of America

## Abstract

**Background:**

In eukaryotic cells, DNA polymerase δ (Polδ), whose catalytic subunit p125 is encoded in the *Pold1* gene, plays a central role in chromosomal DNA replication, repair, and recombination. However, the physiological role of the Polδ in mammalian development has not been thoroughly investigated.

**Methodology/Principal Findings:**

To examine this role, we used a gene targeting strategy to generate two kinds of *Pold1* mutant mice: Polδ-null (*Pold1* ^−/−^) mice and D400A exchanged Polδ (*Pold1* ^exo/exo^) mice. The D400A exchange caused deficient 3′–5′ exonuclease activity in the Polδ protein. In Polδ-null mice, heterozygous mice developed normally despite a reduction in Pold1 protein quantity. In contrast, homozygous *Pold1* ^−/−^ mice suffered from peri-implantation lethality. Although *Pold1* ^−/−^ blastocysts appeared normal, their *in vitro* culture showed defects in outgrowth proliferation and DNA synthesis and frequent spontaneous apoptosis, indicating Polδ participates in DNA replication during mouse embryogenesis. In *Pold1* ^exo/exo^ mice, although heterozygous *Pold1* ^exo/+^ mice were normal and healthy, *Pold1* ^exo/exo^ and *Pold1* ^exo/−^ mice suffered from tumorigenesis.

**Conclusions:**

These results clearly demonstrate that DNA polymerase δ is essential for mammalian early embryogenesis and that the 3′–5′ exonuclease activity of DNA polymerase δ is dispensable for normal development but necessary to suppress tumorigenesis.

## Introduction

DNA replication is the process for transmitting genetic information to future cells and offspring. To date, at least 14 eukaryotic DNA-dependent DNA polymerases have been found [1]–[4]. DNA polymerase α (Polα), DNA polymerase δ (Polδ) and DNA polymerase ε (Polε) are thought to be distinct DNA polymerases that directly participate in DNA replication [3], [5]. These three polymerases belong to the type B DNA polymerase subfamily [6]. Polα forms a Polα/RNA primase complex that synthesizes short RNA-DNA primers for DNA synthesis. On the other hand, Polδ and Polε are believed to extend these short primers; Polδ is mainly responsible for lagging strand synthesis while Polε is thought to be responsible for leading strand synthesis (reviewed in [3], [7]). However, the precise molecular roles of Polδ and Polε remain unclear. Furthermore, in addition to DNA replication, it is known that these elongating polymerases are also involved in several DNA repair and recombination pathways [1], [5].

Our interest is in Polδ, which is a highly processive polymerase that associates with PCNA. The Polδ holoenzyme consists of several subunits: the catalytic subunit p125 (Pold1), which is encoded in the *Pold1* gene and is highly conserved among eukaryotes; and two to four more divergent smaller subunits which are believed to regulate PCNA binding, Polδ function and its structure. Pold1 comprises of two primary functional domains: an exonuclease domain near the N-terminus that catalyzes 3′–5′ exonucleolytic proofreading and the subsequent polymerase domain that catalyzes DNA synthesis.

The molecular functions of Polδ had been revealed mainly by using biochemical analysis and yeast genetics. Studies using the complete replication of DNA plasmids containing the Simian Virus 40 (SV40) DNA replication origin with purified mammalian cell extracts showed that Polδ is required for *in vitro* DNA replication [8], while a study using *Xenopus* egg extracts immunodepleted of Polδ also showed the indispensability of Polδ for DNA replication [9]. In fission yeast, temperature-sensitive mutants and tetrad analysis from diploid heterozygous Pold1-disrupted mutants showed that Pold1 was essential for viability while disruption of Pold1 resulted in a terminal phenotype similar to a cell division cycle mutant, indicating that Polδ was involved in DNA replication [4]. The function of each Pold1 domain has been investigated extensively by using various yeast mutants. For example, mutations disrupting 3′–5′ exonuclease activity caused a strong mutator phenotype in budding yeast [10], [11]. Mutations in motif A of the polymerase domain also expressed a mutator phenotype and hypersensitivity to hydroxyurea and methylmethane sulfonate in budding yeast [12], [13]. Zinc finger domain deletion mutants showed that the zinc finger domain of Pold1 is responsible for binding to the regulatory subunit [14]. However, despite many studies revealing the molecular roles of Polδ, the necessity of Polδ in mammalian cell has not been uncovered by a genetic approach.

Compared to the molecular functions of Polδ in eukaryotic cells, the physiological role of entire Polδ in higher eukaryotes remains obscure. For example, Zebrafish *Pold1* disrupted mutants were found as a result of positional cloning of the *flathead* gene [15]. *Flathead* mutants display specific defects in late proliferative zones, such as eyes, brain and cartilaginous elements of the visceral head skeleton, where cells showed compromised DNA replication. However, the zebrafish mutants did develop normally during their early stages. This may be the result of a functionally redundant gene of the *Pold1* gene due to genomic duplication during teleost evolution. Using mouse genetics, mice deficient in Polδ 3′–5′ exonucelase activity showed that 3′–5′ exonuclease activity suppresses the risk of tumorigenesis as a result of decreasing the spontaneous mutation rate [16], [17]. Mice harboring a mutation in motif A of the polymerase domain were embryonic lethal in homozygotes and experienced genomic instability in heterozygotes as compared to wild types. One motif A mutation, L604K, reduced the life span and accelerated tumorigenesis in heterozygous mice [18]. However, when, how and why these motif A mutant homozygotes suffered from death during embryonic development has not been elucidated well.

In our study, to uncover the physiological role of Polδ in mammalian development more clearly, we generated mice lacking functional Polδ by gene targeting methods in a *Pold1* ^−^ allele and examining its effects. For a more comprehensive analysis, we generated mice lacking 3′–5′ exonuclease activity in the Polδ of a *Pold1* ^exo^ allele. Here, we report that peri-implantation lethality accompanies the loss of functional Polδ. We speculate this is due to an impairment in cell proliferation, a defect in DNA synthesis and an occurrence of apoptosis observed in an *in vitro* blastocyst outgrowth. Polδ is the first DNA polymerase to be studied in such a model offering new insight on how DNA polymerases are involved in nuclear chromosomal replication.

## Results

### Generation of *Pold1* gene targeted mice

**Figure 1:**
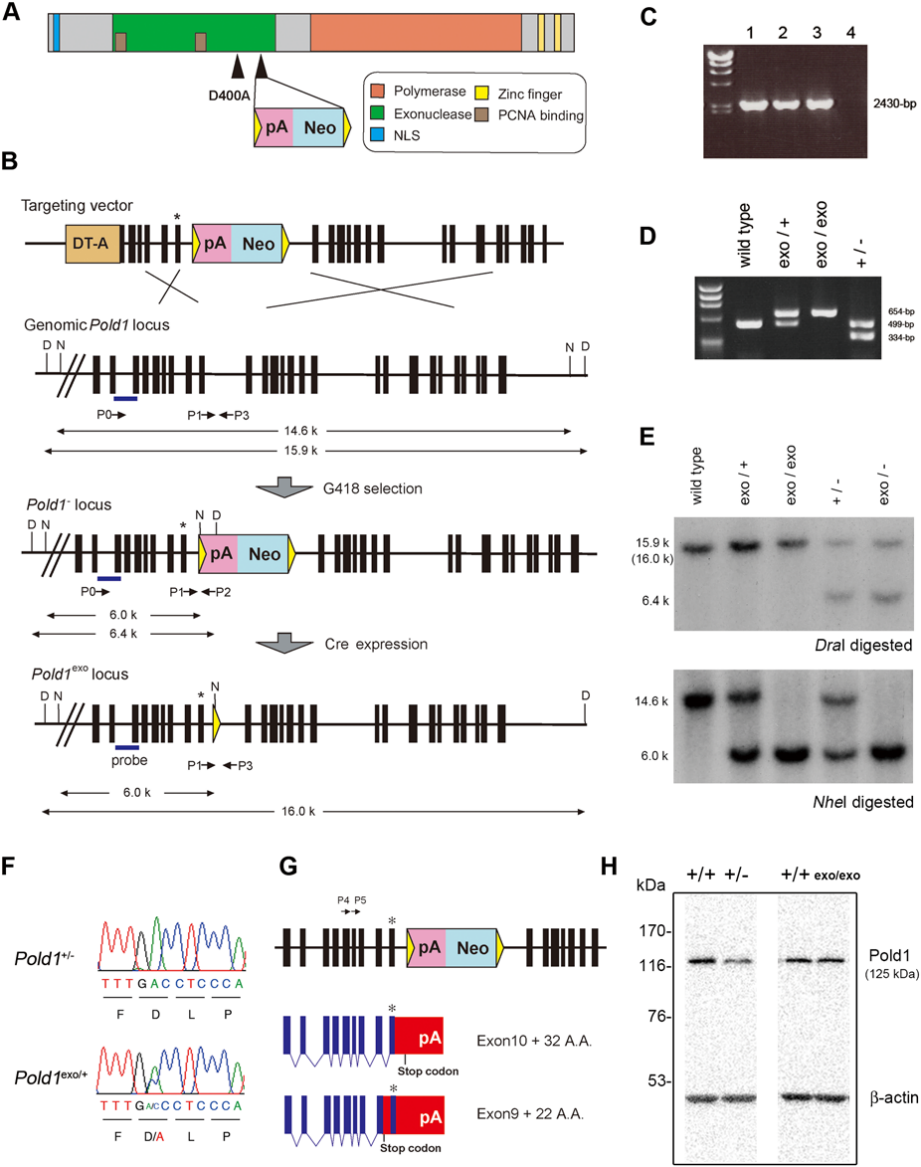
Generation of two kinds of *Pold1* mutated mice by gene targeting strategy. (A) Schematic of the Pold1 domain structure and locations of the modifications with a *Pold1* gene targeting method. (B) Structure of the targeting vector and partial restriction map of the murine *Pold1* locus before and after homologous recombination and Cre mediated recombination. Exons are represented by vertical black boxes and introns by intervening horizontal lines. * indicate D400A substitution. The genomic fragment used as a probe for Southern blotting is indicated by the horizontal blue bar. Restriction enzymes: D indicates *Dra*I; N indicates *Nhe*I. PCR primers P0 and P2 were used to screen for homologous recombinant ES cells. PCR primers P1, P2 and P3 were used for genotyping. (C) PCR screening for homologous recombinants. A 2430-bp band was amplified with the primer pairs P0–P2, specific to the homologous recombinant. Lane 1–3 are independent homologous recombinant ES clones; lane 4 is a wild-type ES clone. (D) PCR genotyping of mice tail DNA. A 499-bp wild-type band and a 654-bp *Pold1* ^exo^ band were amplified with primer pairs P1–P3 and a 334-bp *Pold1* ^−^ band was amplified with primer pairs P1–P2. These primers are shown in (B). (E) Southern blot analysis to identify each *Pold1* allele with mouse tail DNA. In *Dra*I digestion, the 15.9-kb fragment corresponds to the wild-type allele, the 16.0-kb fragment corresponds to the *Pold1* ^exo^ allele (the two overlap) and the 6.4-kb fragment corresponds to the *Pold1* ^−^ allele. In *Nhe*I digestion, the 14.6-kb fragment represents the wild-type allele while the 6.0-kb fragment represents both *Pold1* ^−^ and *Pold1* ^exo^ alleles. (F) DNA sequencing of RT-PCR products from heterozygous *Pold1* ^+/−^ and *Pold1* ^exo/+^ ES cells. Aspartic acid, D, was substituted with alanine, A, in *Pold1* ^exo^ alleles. (G) Schematic represents the results of nucleotide sequencing the 3′RACE products derived from *Pold1* ^−^ alleles in *Pold1* ^+/−^ ES cells. Primers P4 and P5 were used for 3′RACE repeated amplification. 3′RACE products from *Pold1* ^−^ alleles were distinguished by means of sequencing the D400A substitution. (H) Immunoblot analysis of two pairs of extracts derived from E12.5 whole embryos performed with anti-Pold1 and anti-β-actin (control) antibodies; one pair compares *Pold1* ^+/−^ and *Pold1* ^+/+^ from the same litter of embryos while the other compares *Pold1* ^exo/exo^ and *Pold1* ^+/+^ embryos.

To analyze the physiological function of the entire Polδ and its 3′–5′ exonuclease activity, we created two types of *Pold1* mutant mice. The *Pold1* gene encodes the catalytic subunit p125 of the Polδ complex. This subunit contains the DNA polymerase domain and 3′–5′ exonuclease domain. To disrupt the functional murine Pold1 protein, we inserted a splice/polyadenylation (poly(A)) signal derived from SV40 into the upstream region encoding the Polδ polymerase domain (schematic in Fig. 1A). Using a point mutation of GCC from GAC (wild-type), 3′–5′ exonuclease activity deficient mice were created by D400A exchange, which is located in the exonuclease active site (ExoII) and with which mice suffered from higher tumor susceptibility [16], [17]. D400A mutation in mice has been confirmed to cause inhibition of 3′–5′ exonuclease activity by *in vitro* biochemical analysis [17]. We used the Cre-mediated *loxP* recombination system in our gene targeting strategy to generate the two kinds of *Pold1* mutant mice: Polδ-null (*Pold1* ^−/−^) mice and its 3′–5′ exonuclease activity deficient (*Pold1* ^exo/exo^) mice (Fig. 1B) [19].

The *Pold1* gene targeting vector was electroporated into C57BL/6J embryonic stem (ES) cells. G418-resistant clones were screened for homologous recombination by PCR (Fig. 1C) and Southern blotting. A point mutation (D400A) was verified by DNA sequencing. The targeted ES cell clones were microinjected into Balb/c blastocysts to generate chimeric mice which transmitted the targeted allele through the germ line. Chimeras were crossed with C57BL/6J females, yielding *Pold1* ^+/−^ mice. The Polδ 3′–5′ exonuclease activity deficient mice were generated by crossing *Pold1* ^+/−^ mice and Cre expressed transgenic animals which contained a murine *Sycp1* gene promoter-*Cre* transgene that expressed Cre-recombinase in germ-line cells. Both *Pold1* ^+/−^ and *Pold1* ^exo/+^ mice were identified by PCR and Southern blot analysis (Fig. 1D and 1E).

To confirm the expression of the *Pold1* gene from these targeted alleles, we analyzed mRNA from targeted ES cells. The *Pold1* ^exo/+^ ES cells were obtained by electroporating circular *Cre*-*pac* plasmids into *Pold1* ^+/−^ ES cells and selecting adequate clones [20]. RT-PCR was performed on transcripts from *Pold1* ^+/−^ and *Pold1* ^exo/+^ ES cells using two primers that covered the full-length *Pold1* coding region. Direct sequencing of these RT-PCR products showed that substituted alleles (GAC→GCC) were expressed in *Pold1* ^exo/+^ ES cells, but not in *Pold1* ^+/−^ ES cells (Fig. 1F). These results revealed that full-length mRNA was not expressed from the *Pold1* ^−^ allele. To investigate the expression from mutated alleles in detail, we determined transcriptional end sites from *Pold1* ^−^ alleles by 3′ RACE analysis. In 3′RACE products from *Pold1* ^+/−^ ES cells, both full-length *Pold1* coding fragments and truncated fragments were observed; while in the products from *Pold1* ^exo/+^ ES cells, only the full-length fragment was observed. After subcloning these fragments, DNA sequencing was performed. DNA sequencing showed that truncated fragments from *Pold1* ^+/−^ ES cells contained a substituted sequence (GCC), the 3′ terminus was prematurely stopped by the inserted SV40 poly(A) signal sequence, and these truncated fragments consisted of two aberrant splicing forms (Fig. 1G). The full-length fragment sequences from *Pold1* ^+/−^ ES cells only contained the wild-type sequence (GAC) and used an endogenous poly(A) signal sequence from the *Pold1* gene. In *Pold1* ^exo/+^ ES cells, mRNA terminated normally and included both substituted and wild-type sequences (two and three of five subclones, respectively). These results suggest that mRNA terminated by the inserted SV40 poly(A) signal was expressed by the *Pold1* ^−^ allele and the full-length transcript with the D400A (GAC→GCC) substitution was expressed by the *Pold1* ^exo^ allele. To confirm that *Pold1* ^−^ allele was impaired in the expression of the Pold1 protein, we analyzed the Pold1 protein by Western blotting. Western blotting of the extracts prepared from *Pold1* ^+/+^ and *Pold1* ^+/−^ E12.5 whole embryos with an anti-Pold1 C-terminal region antibody showed that the Pold1 protein quantity was reduced to about half in *Pold1* ^+/−^ embryos, while the Pold1 protein quantity from *Pold1* ^+/+^ and *Pold1* ^exo/exo^ E12.5 embryonic extracts were comparable (Fig. 1H). These results suggest that the *Pold1* ^−^ allele did not function properly due to the prematurely terminated polymerase domain and that the *Pold1* ^exo^ allele expressed quantities of Polδ similar to the wild-type allele despite lacking 3′–5′ exonuclease activity.

### Deficiency of Pold1 causes embryonic lethal around peri-implantation

Heterozygous *Pold1* ^+/−^ mice were apparently normal and fertile with no detectable developmental abnormalities over 18 months. No defect in the proliferation of embryonic fibroblasts from these animals was found (data not shown). In contrast, no homozygous *Pold1* ^−/−^ mice were detected among the 256 live births from *Pold1* ^+/−^ intercrosses (Table 1), indicating that one functional *Pold1* allele is sufficient for embryonic and postnatal development, whereas inactivation of both alleles leads to embryonic lethality.

**Table 1:** Genotypes of progeny from *Pold1* ^+/−^ intercrosses.

Age	No. (%) of offspring with genotype			No. (%) resorbed	total
	+/+	+/−	−/−		
Neonate	88 (34)	168 (66)	0 (0)		256
E9.5	8 (24)	13 (38)	0 (0)	13 (38)	34
E7.5	10 (29)	16 (47)	0 (0)	8 (24)	34
E4.5	6 (29)	8 (38)	7 (33)	NA^a^	21
E3.5	10 (25)	18 (45)	12 (30)	NA	40

a NA, not available.

**Figure 2:**
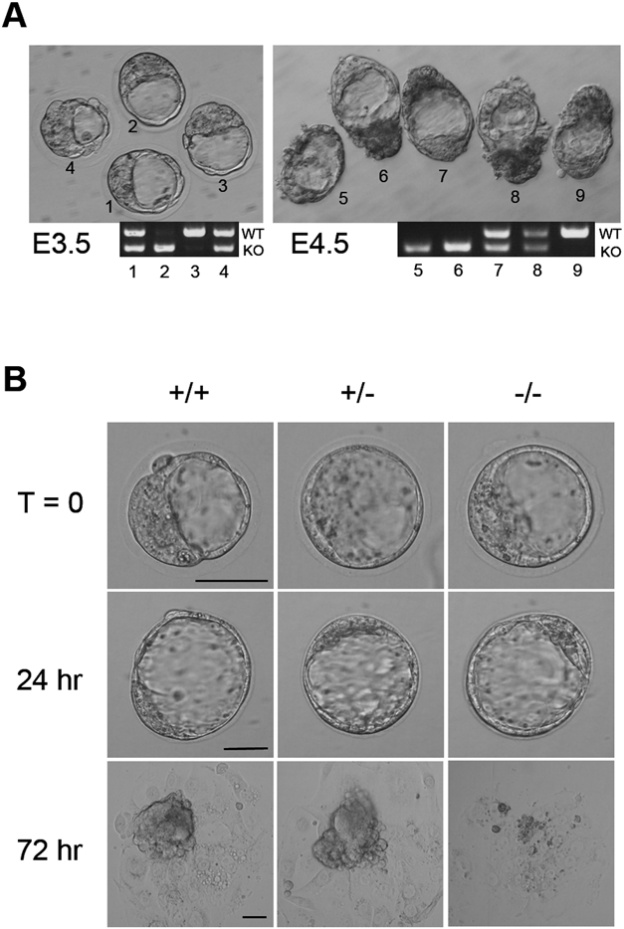
Morphological analysis of blastocysts and outgrowths from blastocysts. (A) The appearance of mutant embryos. E3.5 and E4.5 embryos were flushed out from the uteri. E4.5 embryos were cultured for 2 hr in M2 medium. After taking photographs under bright-field conditions, E3.5 and E4.5 embryos were genotyped by PCR. (B) Impaired proliferation of Pold1-deficient blastocyst outgrowths *in vitro*. Blastocysts were harvested at E3.5, cultured *in vitro* for several days, and subsequently genotyped by PCR. Black horizontal bar indicates 50 µm.

To determine the time of death during the development of *Pold1* ^−/−^ embryos, embryos were collected from intercrosses of *Pold1* ^+/−^ mice at different times of gestation and individual embryos were genotyped by PCR. No homozygous *Pold1*-deficient embryos were found at E7.5 or beyond (Table 1). We also collected embryos at day E3.5 and E4.5 by flushing the uteri of pregnant females. The homozygous *Pold1* ^−/−^ mutant blastocysts were morphologically indistinguishable from wild-type and heterozygous embryos (Fig. 2A). These observations indicate that *Pold1* ^−/−^ embryos died between E4.5 and E7.5.

### Pold1 is required for the proliferation of blastocyst outgrowth

To further characterize the developmental abnormality of Pold1-deficient embryos, we collected E3.5 blastocysts derived from *Pold1* ^+/−^ intercrosses, cultured them *in vitro* individually for several days, took photographs, and subsequently genotyped them by PCR. In *Pold1* ^+/+^ and *Pold1* ^+/−^ blastocysts, trophoblasts spread over the culture dish after hatching from the zona pellucida while an inner cell mass (ICM) grew on the trophoblast sheet after 3 days of culture. In *Pold1* ^−/−^ embryos, the trophoblasts again attached and spread over the dish, but the trophoblast sheet spread slowly and the ICM failed to proliferate and degenerated soon after appearing (Fig. 2B). Pold1-null blastocysts cultured *in vitro* for a day, which roughly corresponded to E4.5 *in vivo*, had no apparent difference from wild-type and heterozygous embryos. *Pold1* ^−/−^ blastocysts cultured for 3 days, roughly corresponding to E6.5, uncovered a serious proliferation defect.

### Lack of Pold1 causes DNA synthesis defects

**Figure 3:**
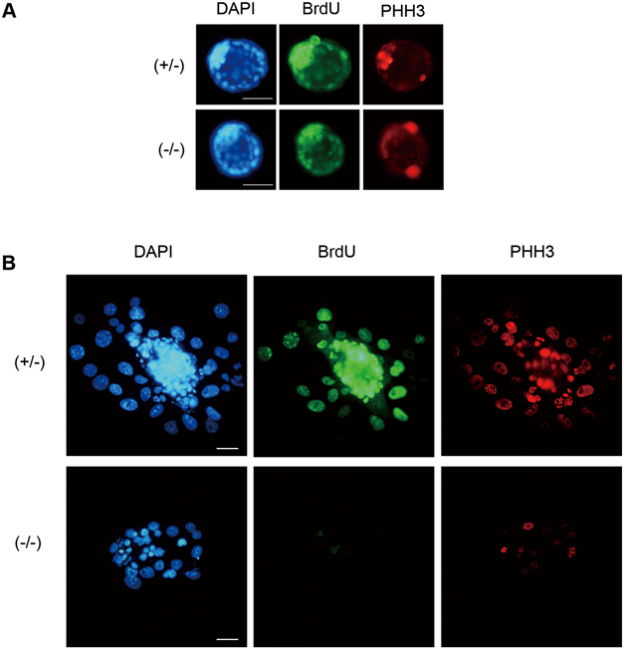
Pold1 deficient embryos showed defective DNA synthesis. DNA synthesis was observed by determining BrdU incorporation in cultured blastocysts *in vitro*. (A) E3.5 embryos were cultured for 24 hr, then cultured in the presence of BrdU for 3 hr. After indirect immunofluorescence using anti-BrdU (green) and anti-pHH3 (red) antibodies and counterstained with DAPI (blue), embryos were genotyped by PCR. (B) E3.5 embryos were cultured for 3 days, cultured in the presence of BrdU for 3 hr, and processed for immunostaining against BrdU (green) and pHH3 (red) and DAPI counterstaining (blue). The genotype of each embryo was subsequently determined by PCR. White horizontal bar indicates 50 µm.

To uncover whether the proliferation defect of the cultured *Pold1* ^−/−^ blastocysts is due to DNA synthesis inhibition caused by Polδ disruption, we assessed DNA synthesis in cultured blastocysts by the bromodeoxyuridine (BrdU) incorporation assay. We cultured blastocysts for 1 or 3 days in normal ES medium and then cultured them in the presence of BrdU for 3 hr. These cultured blastocysts were immunostained with a specific antibody for BrdU. In addition, to assess whether embryonic cells in the cultured *Pold1* ^−/−^ blastocysts entered the mitotic phase, we simultaneously immunostained them with an antibody against phosphohistone H3 (Ser-10), a common mitotic marker used to mark M-phase cells [21]. Incorporation of BrdU into 1 day cultured blastocyst nuclei revealed no differences between each genotype, as all embryos showed extensive labeling (Fig. 3A). We also found phosphohistone H3-positive cells in all embryos regardless of genotype (Fig. 3A). These results suggested that *Pold1* ^−/−^ embryos were able to synthesize new DNA strands, equivalent to the S-phase of the cell cycle, and entered the mitotic phase 1 day after the cultured blastocyst stage. In contrast, in 3 days of cultured blastocyst outgrowth, both the *Pold1* ^+/+^ and *Pold1* ^+/−^ outgrowths showed strong incorporation of BrdU and frequent mitotic stainings into the ICM and trophoblast giant (TG) cells, but *Pold1* ^−/−^ outgrowths showed very little BrdU incorporation and few mitotic cells(Fig. 3B). In TG cells, it has been shown that DNA synthesis occurs by endoreduplication, whereby successive rounds of G and S phases proceed without mitosis [22], [23]. As a result, endocycling giant cells acquire massive quantities of DNA in their nuclei, thereby becoming polyploid [24]. This deficiency in *Pold1* ^−/−^ outgrowths indicates that the disruption of Polδ caused the distinct DNA synthesis defect including the TG cell endoreduplication defect.

### Deficiency of Pold1 protein results in spontaneous apoptosis

**Figure 4:**
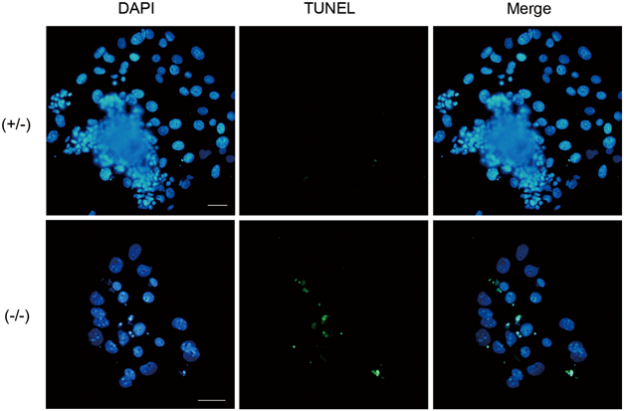
Spontaneous apoptosis detected in Pold1 deficient embryos. Spontaneous apoptosis was analyzed by TUNEL assay. TUNEL-positive cells appeared in Pold1-deficient blastocyst outgrowths. After taking a photo, the genotype of each embryo was determined by PCR. White horizontal bar indicates 50 µm.

In the 3rd day of cultured *Pold1* ^−/−^ outgrowths stained with DAPI, we found condensed and fragmented micronuclei, possibly indicating apoptotic cell death (Fig. 3B). These micronuclei were rarely found in heterozygous or wild-type outgrowths. To confirm this apoptotic possibility, TUNEL assays were performed on blastocyst outgrowths cultured for 3 days. These aberrant nuclei in *Pold1* ^−/−^ outgrowths were all TUNEL-positive, whereas TUNEL-positive cells were rare in *Pold1* ^+/+^ and *Pold1* ^+/−^ outgrowths (Fig. 4). We further explored apoptotic cell death in harvested E3.5 blastocysts. No apparent difference was observed between the *Pold1* ^−/−^ blastocysts (n = 4) and the blastocysts with other genotypes (n = 17) (date not shown). These data indicate that proliferation defects in *Pold1* ^−/−^ outgrowths were, at least in part, due to an occurrence of apoptosis around hatching.

### One 3′–5′ exonuclease deficient *Pold1* ^exo^ allele enables embryonic development, but increases tumor susceptibility

In Polδ 3′–5′ exonuclease activity deficient mice, it has been shown that the mutant allele does not affect embryo viability and that homozygous mutant mice are fertile. However, recessive mutants are more prone to cancer and death [16], [17]. To confirm the physiological role of Polδ 3′–5′ exonuclease activity and to examine an interaction between the *Pold1* ^exo^ allele and the *Pold1* ^−^ allele, we analyzed this exonuclease activity disrupted mice. We found similar observations as those in [16], [17] for *Pold1* ^exo/exo^ and *Pold1* ^exo/+^ mice, as both were able to grow normally and were fertile (Table 2). At embryonic stage E12.5, no morphological differences between *Pold1* ^exo/exo^ and wild-type embryos were found. Moreover *Pold1* ^exo/−^ mice, which were generated by crossing between *Pold1* ^exo/exo^ and *Pold1^+/−^* mice, could give birth and were also apparently normal and fertile (Table 2). These results showed that for mouse development, Polδ 3′–5′ exonuclease activity is dispensable and one *Pold1* ^exo^ allele is sufficient.

**Table 2:** Offspring of 3′–5′ exonuclease deficient mice.

Parents (male×female)	+/+	exo/+	exo/exo	total
*Pold1* ^exo/+^×*Pold1* ^exo/+^	23	39	14	76
*Pold1* ^+/−^×*Pold1* ^exo/exo^	-	9	8	17

**Figure 5:**
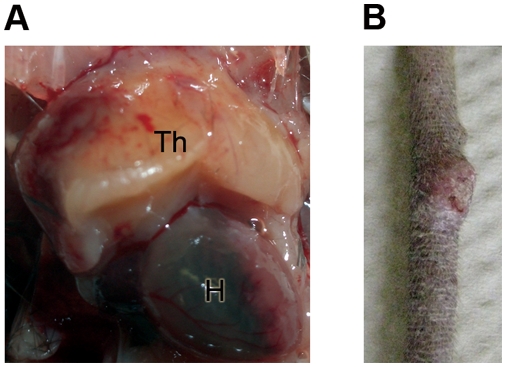
Lack of Polδ 3′–5′ exonuclease activity causes tumors. (A) Swollen thymus in 4-month old *Pold1* ^exo/−^ mice. Th: thymus, H: heart. (B) Tail with nodule in 14-month old *Pold1* ^exo/−^ mice.

Although *Pold1* ^exo/exo^ and *Pold1* ^exo/−^ mice were developmentally normal, they frequently died with abnormally swollen thymuses between 3 and 8 months (9 out of 28 *Pold1* ^exo/exo^ animals and 5 out of 14 *Pold1* ^exo/−^ animals) (Fig. 5A), and many of the surviving mice without swollen thymuses developed nodules on their tails after 12 months (Fig. 5B). These observations are consistent with typical cancer symptoms owing to the lack of Polδ 3′–5′ exonuclease activity [16], [17]. Histological analysis of swollen thymuses showed a homogenous population of cells with a high nuclear to cytoplasmic ratio and frequent mitoses, typical morphological features of lymphoblastic lymphoma (date not shown). *Pold1* ^exo/+^ mice and wild-type mice did not express such phenotypes on their thymus and tails (more than 20 animals observed for each genotype). Thus, the lack of Polδ 3′–5′ exonuclease activity also increased cancer susceptibility in C57BL/6 mice like as those mixed with C57BL/6J and 129/SvJ as previously seen [17]. Furthermore, we found that *Pold1* ^exo/−^ mice developed similar tumors.

## Discussion

So far the function of Polδ had been mainly studied by biochemical approaches and yeast genetics. This present study revealed the function of Polδ by examining its *Pold1* knock out mice. Although many DNA polymerases have been clarified, the physiological role of three DNA polymerases directly involved in chromosomal replication, Polα, Polδ and Polε, have not been assessed in Pol knockout mice. In this study, we found that Polδ disrupted mice suffered from embryonic lethality after E4.5. To date, three DNA polymerases, Polβ [25], [26], Polγ [27] and Polζ [28] are known to lead to embryonic death in mice (after E18.5, E7.5 and E9.5, respectively) upon disruption. Polδ absence caused lethality at an earlier embryonic stage than these aforementioned DNA polymerases. This is consistent with the notion that Polδ has a fundamental role in cellular proliferation.

Genetic studies in yeast [4] and *in vitro* biochemical studies [8], [9] have shown that Polδ is indispensable for nuclear DNA replicative synthesis. However, in this presented study, we found no obvious abnormality in functionally *Pold1*-null mouse embryos before implantation. Since we could count more than 60 nuclei in a one day cultured *Pold1* ^−/−^ blastocyst (data not shown), at least 6 times of cell division were performed between fertilization and the embryonic death. To investigate whether these embryos could proceed with pre-implantation development without Pold1 protein, we performed immunostaining of *Pold1* ^−/−^ blastocysts with anti-Pold1 antibodies. Pold1 protein was not only found in *Pold1* ^+/−^ and *Pold1* ^+/+^ embryos but also in *Pold1* ^−/−^ embryos, which had no apparent cell cycle defect (Fig. S1). In addition, published microarray data indicated that the Pold1 mRNA presents in mouse unfertilized eggs and early embryos where zygotic gene expression have not started [29]. These results suggested that maternal Pold1 stockpiles could support for proceeding pre-implantation development of *Pold1*-null embryos. Similar peri-implantation lethalities have been found when disrupting other components required for nuclear DNA replication such as FEN1 [30], CDC45 [31], and Psf1 [32]. Maternal compensation is also thought to explain why in zebrafish, *Pold1*-null embryos developed to a late embryonic state [15].

*Pold1* ^−/−^ embryos showed a cell proliferation defect and a DNA synthetic defect in *in vitro* blastocyst outgrowths. These observations show that no redundant activity for Polδ exists in mammalian development and affirm the known Polδ cellular function requirement in chromosomal DNA synthesis. Our Polδ results also show frequent spontaneous apoptotic cells and few mitotic cells in 3 day cultured *Pold1* ^−/−^ blastocyst outgrowths (Fig. 3B), indicating the existence of a DNA replication checkpoint in eukaryotic cells to guarantee an accurate transmission of chromosomal DNA to subsequent generations [33]. Disrupting DNA replication is known to activate this checking system, resulting in either arresting the cell cycle before mitosis initiation in order to have the disruption removed or tolerated, or causing apoptosis on cells that harbor the disruption [34]. Our observations in 3 day cultured *Pold1* ^−/−^ blastocyst outgrowths is an expected cellular response to the defective DNA replication caused by Polδ disruption.

Although heterozygous *Pold1* ^+/−^ mice showed a reduction in the amount of Pold1 protein (Fig. 1H), there were no apparent developmental abnormalities. In addition, *Pold1* ^−/−^ embryos developed normally until the blastocyst stage despite zygotic gene expression starting at the two cell stage. These observations showed that *in vivo*, the *Pold1* gene expresses more than the necessary amount of gene products needed for development and indicate that the gross quantity of Pold1 protein does not regulate the mammalian developmental process.

In contrast to the functional disruption seen with the *Pold1* gene, it has been seen that homozygous deficient (*Pold1* ^exo/exo^) mice for Polδ 3′–5′ exonuclease activity develop normally, although they are more susceptible to tumors [16], [17]. We found similar phenotypes in *Pold1* ^exo/−^ mice. Similar types of tumors were observed in both *Pold1* ^exo/exo^ and *Pold1* ^exo/−^ mice lacking exonuclease activity. The tumorigenesis in *Pold1* ^exo/−^ mice revealed more clearly that one *Pold1* ^exo^ allele was sufficient for increasing cancer susceptibility without *Pold1* ^+^ allele and Polδ 3′–5′ exonuclease activity expressed a dominant effect on the suppression of tumorigenesis.

Most of our knowledge about DNA replication has accumulated from biochemical studies and genetic studies on lower eukaryotes. But these models do not necessarily correlate well with higher eukaryotic systems because eukaryotic systems have many more types of differentiated cells with more complicated interactions. For example, a study of DNA polymerase genes during the development of mouse testis suggested that Polε did not participate in meiotic replication, though it is thought that this polymerase cooperates with two others, Polα and Polδ, in DNA replication based on other models [35]. Additionally, reliability of DNA replication, in which Polδ is involved, affects cancer susceptibility in higher eukaryotes.

Our present findings revealed the physiological functions of Polδ during mammalian development by using *Pold1* gene targeted mice, indicating that the molecular function of Polδ is conserved at least in mammalian early embryogenesis and that its 3′–5′ exonuclease activity is necessary for tumor suppression. Further analysis on the physiological function of replicative DNA polymerases will shed light on the mechanism of DNA replication in mammalian systems, adding critical insight to genomic instability.

## Materials and Methods

### Animals

Animals were maintained in a specific pathogen-free space under a 12-h light/dark regime with access to food and water *ad libitum*. Experimental procedures were in accordance with the Guide for the Care and Use of Laboratory Animals of the Science Council of Japan and were approved by the Animal Experiment Committee of Osaka University.

### Construction of the gene targeting vector

The targeting vector was cloned into pBlueScriptII (Invitrogen) and consisted of four fragments. The bacterial artificial chromosome (BAC) clone RP23-406H21 comprised the murine genomic *Pold1* coding region. For homologous recombination, a 3′ fragment (6.9-kb) from the intron between exons 10 and 11 to exon 25 was extracted with *Eco*RI and *Sac*I digestion from the BAC clone. The 5′ fragment (1.6-kb) from exon 5 to the intron between exons 10 and 11 contained a mutation which changes the D400 codon to an alanine codon. This mutated fragment was generated by repeated PCR using the oligonucleotide 5′-CAGAACTTTGCCCTCCCATACCTC-3′ and its complementary oligonucleotide (alanine codon is underlined). Floxed fragments, which contained the 800-bp simian virus 40 (SV40) splice/polyadenylylation signal [36] and a neomycin resistance cassette for positive selection, were inserted between the 5′ and 3′ fragments for homologous recombination and in the same orientation relative to the *Pold1* gene. A DT-A fragment for negative selection was placed outside the 5′ fragment for homologous recombination [37]. The targeting vector was verified by restriction digestion and nucleotide sequencing.

### Generation of *Pold1* gene targeting mice

The *Sac*I-linearized targeting vector was electroporated into C57BL/6J embryonic stem (ES) cells (InGenious targeting, New York). Candidate recombinant clones were selected by growth in the presence of G418. Adequate homologous recombinants were verified by PCR, Southern blotting and DNA sequencing. For PCR, primers P0 (5′-TTGACCTCCGCACTCATCAG-3′) and P2 (5′-CACCAGACCAACTGGTAATGG-3′) were used with *LA Taq* polymerase (TaKaRa). For Southern blotting, genomic DNA was completely digested by *Dra*I or *Nhe*I, and probed with external [^32^P]dCTP-labeled DNA fragments as indicated in Fig. 1B. Injection of the recombinant ES cells into Balb/c blastocysts generated chimeric mice. The chimeras were mated with C57BL/6J female mice to generate heterozygous *Pold1* ^+/−^ mice. The *Pold1* ^exo/+^ mice were generated by crossing *Pold1* ^+/−^ mice and Sycp1-Cre transgenic mice. All mutations used in this study maintained on the inbred (C57BL/6) background. For genotyping these *Pold1* targeting mice, PCRs were performed with three primers: P1 (5′-GGAGTCCAGGTGTGCGTTAC-3′), P2 (5′-CACCAGACCAACTGGTAATGG-3′) and P3 (5′-CAGATTCCCCTCTGTGCATC-3′). PCR was done at 94°C for 1 min, followed by 30 cycles at 94°C for 1 min, 60°C for 30 s, and 72°C for 40 s with *EX Taq* polymerase (TaKaRa).

### Establishment of *Pold1* ^exo/+^ ES cell lines

To obtain *Pold1* ^exo/+^ ES cells, circular pCre-Pac plasmids were electroporated into *Pold1* ^+/−^ ES cells [20]. The candidate ES cells were selected transiently with puromycin (1 µg/ml; Sigma). Each genotype of these Cre-mediated recombinants was confirmed by PCR and Southern blotting.

### Generation of Sycp1-Cre Tg mice

The plasmid to create Sycp1-Cre Tg mice was constructed from two fragments. One fragment was the promoter sequence from the murine *Sycp1* gene, which expressed in male germ cells, including the region from −737 to +87 relative to the transcription start site [38]. The other was the *Cre* coding sequence containing the nuclear localized signal and polyadenylation signal. Both fragments were inserted into the pBluescript II (Stratagene) between the *Spe*I and *Hind*III sites. Then the plasmid was digested by *Sal*I and *Not*I and the purified fragment was microinjected into fertilized eggs derived from C57BL/6 mice. We screened the offspring for an insertion of the transgene by genomic PCR using the Cre specific primers (5′-CTGAGAGTGATGAGGTTC-3′) and (5′-CTAATCGCCATCTTCCAGCAG-3′).

### RT-PCR and 3′RACE analysis

Total RNA was isolated from ES cells using TRIzol Reagent (GIBCO) and digested with DNaseI to remove contaminating genomic DNA. cDNA was prepared by using Superscript III Reverse Transcriptase (Invitrogen) with an oligo(dT)_25_ primer. PCR was performed with the *Pold1* 5′-terminal primer (5′-GGCGTATCTTGTGGCGGGAA-3′) and 3′-terminal primer (5′-CCTTGTCCCGTGTCAGGTCA-3′). For 3′RACE, reverse transcription was performed with an adaptor sequence conjugated to an oligo(dT)_17_ primer while the subsequently repeated PCRs used primers P4 (5′-TCATGGCCCTTCTCCATTTC-3′), P5 (5′-TGGAGCTGCCAGCTGGAAAG-3′) (Fig. 1G) and two kinds of adaptor primers.

### Western blotting

E12.5 whole embryos were lysed in ice-cold lysis buffer (0.1% NP-40, 50 mM Tris-HCl [pH 7.2], 250 mM NaCl, 2 mM EDTA, 10% glycerol, 1 mM PMSF, 3 µg/ml leupeptine, 3 µg/ml pepstatineA, 10 µg/ml aprotinin), homogenized with Polytron and then sonicated briefly. The lysates were cleared by centrifugation at 20,000×g for 20 min. The supernatants were collected and used for Western blotting. For Western blotting, anti-Pold1 C-terminal region polyclonal antibody (C-20, Santa Cruz) and anti-β-actin monoclonal antibody (AC-15, Sigma) were used.

### Blastocyst culture and genotyping

All embryos were generated by natural mating. The morning of the day on which the vaginal plug was detected was designated as day E0.5. Embryos were collected on E3.5 or E4.5 by flushing the uteri with M2 medium (Sigma). For culture, embryos were then cultured in complete ES cell medium containing leukemia inhibitory factor. In BrdU treated embryos, embryos were additionally cultured in the ES cell medium supplemented with 10 µM BrdU for 3 hours.

For genotyping, individual embryos were lysed by incubation at 55°C overnight in 5 µl PCR lysis buffer (10 mM Tris-HCl [pH 8.0], 50 mM KCl, 2 mM MgCl_2_, 0.45% NP-40, 0.45% Tween 20, 0.2 mg/ml of proteinase K) [39]. To detect each mutated allele, PCRs were performed with the same three primers as mice genotyping: P1, P2 and P3. PCR was done at 94°C for 1 min, followed by 35 cycles at 94°C for 1 min, 58°C for 30 s, and 72°C for 50 s with *LA Taq* polymerase (TaKaRa).

### Immunocytochemistry and apoptotic cell detection

Embryos were washed in phosphate-buffered saline (PBS) containing 1.5% bovine serum albumin (BSA), fixed in 4% paraformaldehyde in PBS for 30 min at 4°C, and then permeabilized for 20 min at room temperature in PBS with 0.3% Triton X-100 and 1.5% BSA. For BrdU-treated embryos, DNA was denatured after permeabilization with 0.25 N HCl and 0.5% Triton X-100 for 20 min at room temperature and washed extensively in PBS with 1.5% BSA. Embryos were incubated with specific primary antibodies overnight at 4°C. The primary antibodies used in this study were mouse anti-BrdU (Dako Cytomation) and rabbit anti-phosphohistone H3 (Ser-10) (Cell Signaling). The fluorescence-labeled secondary antibodies were purchased from Molecular Probes. TUNEL (terminal deoxynucleotidyltransferase-mediated dUTP-biotin nick end labeling) assays to detect apoptotic cells were performed by using the In Situ Cell Death Detection Kit (Roche).

## Supporting Information

Figure S1 Pold1 protein exists in *Pold1* ^−/−^ blastocysts. To check if *Pold1* gene products are present in *Pold1* deficient embryos, we immunostained blastocysts harvested from *Pold1* ^+/−^ intercross using two kinds of anti-Pold1 polyclonal antibodies, H300 and C20 (purchased from Santa Cruz) and counterstained with DAPI. After taking a photo, the genotype of each embryo was determined by PCR. White horizontal bar indicates 50 µm.
(0.73 MB TIF)

## References

[1] Pavlov YI, Shcherbakova PV, Rogozin IB (2006). Roles of DNA polymerases in replication, repair, and recombination in eukaryotes.. Int Rev Cytol.

[2] Burgers PM, Koonin EV, Bruford E, Blanco L, Burtis KC (2001). Eukaryotic DNA polymerases: proposal for a revised nomenclature.. J Biol Chem.

[3] Garg P, Burgers PM (2005). DNA polymerases that propagate the eukaryotic DNA replication fork.. Crit Rev Biochem Mol Biol.

[4] Francesconi S, Park H, Wang TS (1993). Fission yeast with DNA polymerase delta temperature-sensitive alleles exhibits cell division cycle phenotype.. Nucleic Acids Res.

[5] Hubscher U, Maga G, Spadari S (2002). Eukaryotic DNA polymerases.. Annu Rev Biochem.

[6] Braithwaite DK, Ito J (1993). Compilation, alignment, and phylogenetic relationships of DNA polymerases.. Nucleic Acids Res.

[7] Pursell ZF, Isoz I, Lundstrom EB, Johansson E, Kunkel TA (2007). Yeast DNA polymerase epsilon participates in leading-strand DNA replication.. Science.

[8] Waga S, Stillman B (1998). The DNA replication fork in eukaryotic cells.. Annu Rev Biochem.

[9] Fukui T, Yamauchi K, Muroya T, Akiyama M, Maki H (2004). Distinct roles of DNA polymerases delta and epsilon at the replication fork in Xenopus egg extracts.. Genes Cells.

[10] Simon M, Giot L, Faye G (1991). The 3′ to 5′ exonuclease activity located in the DNA polymerase delta subunit of Saccharomyces cerevisiae is required for accurate replication.. EMBO J.

[11] Morrison A, Johnson AL, Johnston LH, Sugino A (1993). Pathway correcting DNA replication errors in Saccharomyces cerevisiae.. EMBO J.

[12] Li L, Murphy KM, Kanevets U, Reha-Krantz LJ (2005). Sensitivity to phosphonoacetic acid: a new phenotype to probe DNA polymerase delta in Saccharomyces cerevisiae.. Genetics.

[13] Venkatesan RN, Hsu JJ, Lawrence NA, Preston BD, Loeb LA (2006). Mutator phenotypes caused by substitution at a conserved motif A residue in eukaryotic DNA polymerase delta.. J Biol Chem.

[14] Sanchez Garcia J, Ciufo LF, Yang X, Kearsey SE, MacNeill SA (2004). The C-terminal zinc finger of the catalytic subunit of DNA polymerase delta is responsible for direct interaction with the B-subunit.. Nucleic Acids Res.

[15] Plaster N, Sonntag C, Busse CE, Hammerschmidt M (2006). p53 deficiency rescues apoptosis and differentiation of multiple cell types in zebrafish flathead mutants deficient for zygotic DNA polymerase delta1.. Cell Death Differ.

[16] Goldsby RE, Lawrence NA, Hays LE, Olmsted EA, Chen X (2001). Defective DNA polymerase-delta proofreading causes cancer susceptibility in mice.. Nat Med.

[17] Goldsby RE, Hays LE, Chen X, Olmsted EA, Slayton WB (2002). High incidence of epithelial cancers in mice deficient for DNA polymerase delta proofreading.. Proc Natl Acad Sci U S A.

[18] Venkatesan RN, Treuting PM, Fuller ED, Goldsby RE, Norwood TH (2007). Mutation at the polymerase active site of mouse DNA polymerase delta increases genomic instability and accelerates tumorigenesis.. Mol Cell Biol.

[19] Sauer B (1998). Inducible gene targeting in mice using the Cre/lox system.. Methods.

[20] Taniguchi M, Sanbo M, Watanabe S, Naruse I, Mishina M (1998). Efficient production of Cre-mediated site-directed recombinants through the utilization of the puromycin resistance gene, pac: a transient gene-integration marker for ES cells.. Nucleic Acids Res.

[21] Hendzel MJ, Wei Y, Mancini MA, Van Hooser A, Ranalli T (1997). Mitosis-specific phosphorylation of histone H3 initiates primarily within pericentromeric heterochromatin during G2 and spreads in an ordered fashion coincident with mitotic chromosome condensation.. Chromosoma.

[22] Barlow PW, Sherman MI (1972). The biochemistry of differentiation of mouse trophoblast: studies on polyploidy.. J Embryol Exp Morphol.

[23] Gardner RL (1983). Origin and differentiation of extraembryonic tissues in the mouse.. Int Rev Exp Pathol.

[24] Varmuza S, Prideaux V, Kothary R, Rossant J (1988). Polytene chromosomes in mouse trophoblast giant cells.. Development.

[25] Gu H, Marth JD, Orban PC, Mossmann H, Rajewsky K (1994). Deletion of a DNA polymerase beta gene segment in T cells using cell type-specific gene targeting.. Science.

[26] Sugo N, Aratani Y, Nagashima Y, Kubota Y, Koyama H (2000). Neonatal lethality with abnormal neurogenesis in mice deficient in DNA polymerase beta.. EMBO J.

[27] Hance N, Ekstrand MI, Trifunovic A (2005). Mitochondrial DNA polymerase gamma is essential for mammalian embryogenesis.. Hum Mol Genet.

[28] Wittschieben J, Shivji MK, Lalani E, Jacobs MA, Marini F (2000). Disruption of the developmentally regulated Rev3l gene causes embryonic lethality.. Curr Biol.

[29] Hamatani T, Carter MG, Sharov AA, Ko MS (2004). Dynamics of global gene expression changes during mouse preimplantation development.. Dev Cell.

[30] Larsen E, Gran C, Saether BE, Seeberg E, Klungland A (2003). Proliferation failure and gamma radiation sensitivity of Fen1 null mutant mice at the blastocyst stage.. Mol Cell Biol.

[31] Yoshida K, Kuo F, George EL, Sharpe AH, Dutta A (2001). Requirement of CDC45 for postimplantation mouse development.. Mol Cell Biol.

[32] Ueno M, Itoh M, Kong L, Sugihara K, Asano M (2005). PSF1 is essential for early embryogenesis in mice.. Mol Cell Biol.

[33] Bartek J, Lukas C, Lukas J (2004). Checking on DNA damage in S phase.. Nat Rev Mol Cell Biol.

[34] Roos WP, Kaina B (2006). DNA damage-induced cell death by apoptosis.. Trends Mol Med.

[35] Kamel D, Mackey ZB, Sjoblom T, Walter CA, McCarrey JR (1997). Role of deoxyribonucleic acid polymerase epsilon in spermatogenesis in mice.. Biol Reprod.

[36] Ornitz DM, Moreadith RW, Leder P (1991). Binary system for regulating transgene expression in mice: targeting int-2 gene expression with yeast GAL4/UAS control elements.. Proc Natl Acad Sci U S A.

[37] Yagi T, Nada S, Watanabe N, Tamemoto H, Kohmura N (1993). A novel negative selection for homologous recombinants using diphtheria toxin A fragment gene.. Anal Biochem.

[38] Vidal F, Sage J, Cuzin F, Rassoulzadegan M (1998). Cre expression in primary spermatocytes: a tool for genetic engineering of the germ line.. Mol Reprod Dev.

[39] Le Cam L, Lacroix M, Ciemerych MA, Sardet C, Sicinski P (2004). The E4F protein is required for mitotic progression during embryonic cell cycles.. Mol Cell Biol.

