# Supplementary figures and images for: DNA Polymerase δ Is Required for Early Mammalian Embryogenesis
Source: PLoS One. 2009 Jan 15;4(1):e4184. doi: 10.1371/journal.pone.0004184 (PMC2615215; doi:10.1371/journal.pone.0004184)

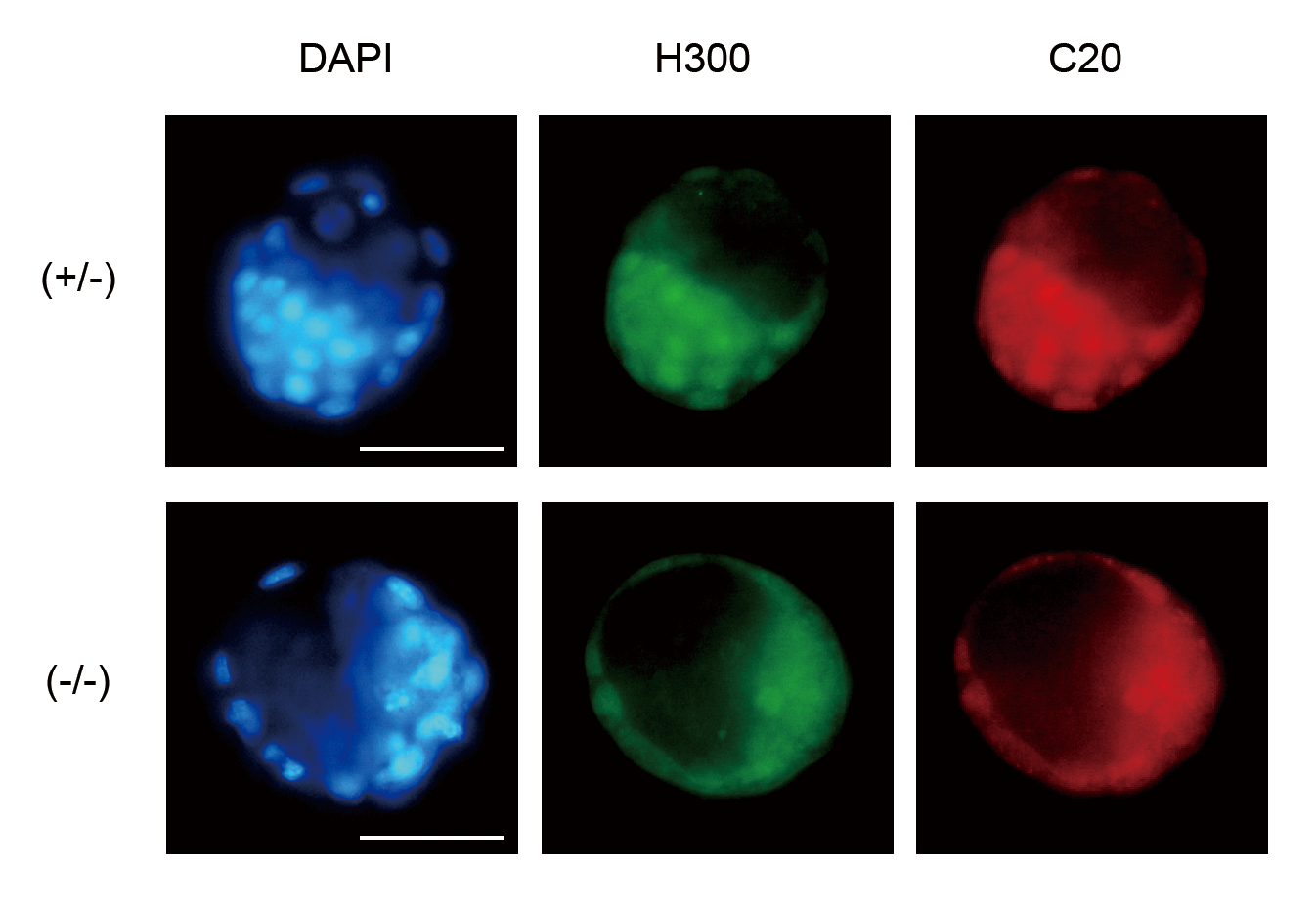

Supplement: Figure S1 — Pold1 protein exists in Pold1 −/− blastocysts. To check if Pold1 gene products are present in Pold1 deficient embryos, we immunostained blastocysts harvested from Pold1 +/− intercross using two kinds of anti-Pold1 polyclonal antibodies, H300 and C20 (purchased from Santa Cruz) and counterstained with DAPI. After taking a photo, the genotype of each embryo was determined by PCR. White horizontal bar indicates 50 µm. (0.73 MB TIF) [file pone.0004184.s001.tif]
